# Association of dietary and nutritional factors with cognitive decline, dementia, and depressive symptomatology in older individuals according to a neurogenesis-centred biological susceptibility to brain ageing

**DOI:** 10.1093/ageing/afae042

**Published:** 2024-05-15

**Authors:** Andrea Du Preez, Sophie Lefèvre-Arbogast, Raúl González-Domínguez, Vikki Houghton, Chiara de Lucia, Hyunah Lee, Dorrain Y Low, Catherine Helmer, Catherine Féart, Cécile Delcourt, Cécile Proust-Lima, Mercè Pallàs, Alex Sánchez-Pla, Mireia Urpi-Sardà, Silvie R Ruigrok, Barbara Altendorfer, Ludwig Aigner, Paul J Lucassen, Aniko Korosi, Claudine Manach, Cristina Andres-Lacueva, Cécilia Samieri, Sandrine Thuret

**Affiliations:** Department of Basic and Clinical Neuroscience, Maurice Wohl Clinical Neuroscience Institute, Institute of Psychiatry, Psychology and Neuroscience, King’s College London, London SE5 9NU, UK; University of Bordeaux, Inserm, Bordeaux Population Health Research Center, UMR 1219, F-33000 Bordeaux, France; Nutrition, Food Science and Gastronomy Department, Faculty of Pharmacy and Food Science, University of Barcelona, 08028 Barcelona, Spain; CIBER Fragilidad y Envejecimiento Saludable (CIBERFES), Instituto de Salud Carlos III, 0828 Barcelona, Spain; Department of Basic and Clinical Neuroscience, Maurice Wohl Clinical Neuroscience Institute, Institute of Psychiatry, Psychology and Neuroscience, King’s College London, London SE5 9NU, UK; Department of Basic and Clinical Neuroscience, Maurice Wohl Clinical Neuroscience Institute, Institute of Psychiatry, Psychology and Neuroscience, King’s College London, London SE5 9NU, UK; Department of Basic and Clinical Neuroscience, Maurice Wohl Clinical Neuroscience Institute, Institute of Psychiatry, Psychology and Neuroscience, King’s College London, London SE5 9NU, UK; INRA, Human Nutrition Unit, UMR1019, Université Clermont Auvergne, F-63000 Clermont Ferrand, France; University of Bordeaux, Inserm, Bordeaux Population Health Research Center, UMR 1219, F-33000 Bordeaux, France; University of Bordeaux, Inserm, Bordeaux Population Health Research Center, UMR 1219, F-33000 Bordeaux, France; University of Bordeaux, Inserm, Bordeaux Population Health Research Center, UMR 1219, F-33000 Bordeaux, France; University of Bordeaux, Inserm, Bordeaux Population Health Research Center, UMR 1219, F-33000 Bordeaux, France; Pharmacology Section, Department of Pharmacology, Toxicology and Medicinal Chemistry, Faculty of Pharmacy and Food Sciences, and Institute of Neurosciences, University of Barcelona, E-08028 Barcelona, Spain; Nutrition, Food Science and Gastronomy Department, Faculty of Pharmacy and Food Science, University of Barcelona, 08028 Barcelona, Spain; CIBER Fragilidad y Envejecimiento Saludable (CIBERFES), Instituto de Salud Carlos III, 0828 Barcelona, Spain; Nutrition, Food Science and Gastronomy Department, Faculty of Pharmacy and Food Science, University of Barcelona, 08028 Barcelona, Spain; CIBER Fragilidad y Envejecimiento Saludable (CIBERFES), Instituto de Salud Carlos III, 0828 Barcelona, Spain; Brain Plasticity Group, Swammerdam Institute for Life Sciences, Center for Neuroscience, University of Amsterdam, 1098 XH Amsterdam, The Netherlands; Institute of Molecular Regenerative Medicine, Spinal Cord Injury and Tissue Regeneration Center Salzburg, Paracelsus Medical University, Salzburg 5020, Austria; Institute of Molecular Regenerative Medicine, Spinal Cord Injury and Tissue Regeneration Center Salzburg, Paracelsus Medical University, Salzburg 5020, Austria; Brain Plasticity Group, Swammerdam Institute for Life Sciences, Center for Neuroscience, University of Amsterdam, 1098 XH Amsterdam, The Netherlands; Brain Plasticity Group, Swammerdam Institute for Life Sciences, Center for Neuroscience, University of Amsterdam, 1098 XH Amsterdam, The Netherlands; INRA, Human Nutrition Unit, UMR1019, Université Clermont Auvergne, F-63000 Clermont Ferrand, France; Nutrition, Food Science and Gastronomy Department, Faculty of Pharmacy and Food Science, University of Barcelona, 08028 Barcelona, Spain; CIBER Fragilidad y Envejecimiento Saludable (CIBERFES), Instituto de Salud Carlos III, 0828 Barcelona, Spain; University of Bordeaux, Inserm, Bordeaux Population Health Research Center, UMR 1219, F-33000 Bordeaux, France; Department of Basic and Clinical Neuroscience, Maurice Wohl Clinical Neuroscience Institute, Institute of Psychiatry, Psychology and Neuroscience, King’s College London, London SE5 9NU, UK; Department of Neurology, University Hospital Carl Gustav Carus, Technische Universität Dresden, 01307 Dresden, Germany

**Keywords:** diet, hippocampal neurogenesis, cognitive decline, dementia, late-life depression, older people

## Abstract

Hippocampal neurogenesis (HN) occurs throughout the life course and is important for memory and mood. Declining with age, HN plays a pivotal role in cognitive decline (CD), dementia, and late-life depression, such that altered HN could represent a neurobiological susceptibility to these conditions. Pertinently, dietary patterns (e.g., Mediterranean diet) and/or individual nutrients (e.g., vitamin D, omega 3) can modify HN, but also modify risk for CD, dementia, and depression. Therefore, the interaction between diet/nutrition and HN may alter risk trajectories for these ageing-related brain conditions. Using a subsample (*n* = 371) of the Three-City cohort—where older adults provided information on diet and blood biobanking at baseline and were assessed for CD, dementia, and depressive symptomatology across 12 years—we tested for interactions between food consumption, nutrient intake, and nutritional biomarker concentrations and neurogenesis-centred susceptibility status (defined by baseline readouts of hippocampal progenitor cell integrity, cell death, and differentiation) on CD, Alzheimer’s disease (AD), vascular and other dementias (VoD), and depressive symptomatology, using multivariable-adjusted logistic regression models. Increased plasma lycopene concentrations (OR [95% CI]  =  1.07 [1.01, 1.14]), higher red meat (OR [95% CI]  =  1.10 [1.03, 1.19]), and lower poultry consumption (OR [95% CI]  =  0.93 [0.87, 0.99]) were associated with an increased risk for AD in individuals with a neurogenesis-centred susceptibility. Increased vitamin D consumption (OR [95% CI]  =  1.05 [1.01, 1.11]) and plasma γ-tocopherol concentrations (OR [95% CI]  =  1.08 [1.01, 1.18]) were associated with increased risk for VoD and depressive symptomatology, respectively, but only in susceptible individuals. This research highlights an important role for diet/nutrition in modifying dementia and depression risk in individuals with a neurogenesis-centred susceptibility.

## Key Points

- Adult hippocampal neurogenesis (HN) plays a key role in the pathogenesis of cognitive decline (CD), dementia, and depression.
- HN could even represent a neurobiological susceptibility to these conditions.
- Diet not only modifies the risk for CD, dementia, and depression but also modifies HN.
- We show here that the interaction between diet and HN can modify the risk trajectories of these ageing-related brain conditions.
- Further research is required to fully understand the impact of diet on the risk trajectories of susceptible individuals.

## Introduction

Ageing is a highly diverse experience marked by significant disparities in brain health. In particular, the prevalence of cognitive decline (CD), dementia, and late-life depression (LLD) all increase significantly with age [1]. With life expectancy continuing to rise, the impact of these conditions will only become more burdensome. Therefore, to identify more effective healthy brain ageing strategies and interventions [2, 3], we need to deepen our understanding of the factors that promote or hinder these ageing-related brain conditions.

Several risk factors have been implicated in the development of CD, dementia, and LLD but evidence suggests that adult hippocampal neurogenesis (HN; the birth of new neurons in the adult brain [4]) plays a pivotal role in the pathogenesis of all three of these conditions [5–8]. In this regard, we recently demonstrated how differences in HN are associated with CD, dementia, and depressive symptomatology 12 years later [9, 10], indicating that altered HN could represent a neurobiological susceptibility to these conditions.

Importantly, we and others have also shown how diet not only modifies the risk for CD, dementia, and LLD [11–15] but also the neurogenic process [16, 17]. Therefore, it stands to reason that diet and HN could interact, modifying the risk trajectories of CD, dementia, and LLD. Thus, leveraging data from our previous work on a subsample of the Three-City (3C) prospective cohort [9, 10], we aimed to test whether diet could modify the risk for future CD, dementia, and depressive symptomatology in those with and without a neurogenesis-centred biological susceptibility.

## Methods and materials

### Population and study design

**Figure 1:**
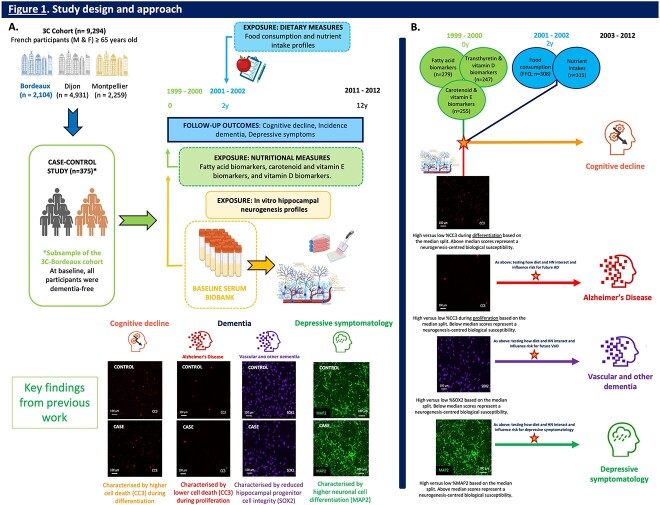
**Study design and approach**. (A) Three City (3C) cohort and sample: Participants from the 3C study (*n* = 9,294) were recruited from three French cities: Bordeaux (*n* = 2,104), Dijon (*n* = 4,931) and Montpellier (*n* = 2,259) and specifically, a case–control study design on cognitive decline (CD) status (*n* = 375), nested within the 3C-Bordeaux cohort, was used for present analyses. Exposures: (i) Neurogenesis-centred biological susceptibility: Our previous work indicated that there may be a neurogenesis-centred biological susceptibility for CD, dementia, and depressive symptomatology that is already present up to 12 years prior to condition onset [9, 10]. Specifically, we found that increased baseline levels of cell death during differentiation (i.e., %CC3-d) increased the risk for future CD, whereas decreased baseline levels of cell death during proliferation (i.e., %CC3-p) increased the risk for future AD. Additionally, we found that reduced baseline levels of hippocampal progenitor cell integrity (i.e. %SOX2) increased the risk for VoD, whereas increased hippocampal cell differentiation (i.e., %MAP2) increased the risk for depressive symptomatology. Therefore, for present analyses, we categorised all participants with hippocampal neurogenesis (HN) profiles (*n* = 371), using a dichotomous classification approach (median split), focusing on biological susceptibility centred around these key HN readouts. (ii) Diet and nutrition: Data from three dietary/nutritional aspects were used to inform present analyses: (i) nutritional biomarker concentrations, including 12 fatty acids (*n* = 279), transthyretin and vitamin D (*n* = 247), and 6 carotenoid and 3 vitamin E biomarkers (*n* = 255), (ii) food consumption (in servings per week, *n* = 308), and (iii) macro- and micronutrient intakes (*n* = 315). Nutritional biomarker concentrations were measured in total plasma collected at baseline. Food consumption and nutrient intakes were determined by the Food Frequency Questionnaire (FFQ) and 24-h dietary recall, respectively, and were collected at the 2-year follow-up. (ii) Outcomes: (i) CD*:* Participants were classified as either having cognitive stability (control) or accelerated CD (case) based on their cognitive trajectories over 12 years. Cases had the worst slopes of CD across follow-up, whereas controls maintained cognitive function above the median slope. (ii) Dementia: At baseline, all participants were dementia-free. Over 12 years, dementia diagnosis was established by an independent committee of neurologists, following Diagnostic and Statistical Manual of Mental Disorders IV criteria. Dementia subtypes were consolidated into two primary categories for analysis, because of limited case numbers, which encompassed Alzheimer's Disease (AD) (i.e., probable/possible AD and mixed dementia) or VoD (i.e. vascular dementia, Parkinson dementia, Lewy body dementia, and frontotemporal dementia). (iii) Depressive symptomatology: Depressive symptomatology was assessed using the Center for Epidemiologic Studies Depression (CES-D) scale. Clinically relevant depressive symptoms at any assessment during the study duration were defined as scores ≥17 in men and ≥23 in women, or if participants were diagnosed with depression. (B) Overall approach: To determine whether diet and nutrition could influence the risk for future CD, dementia,and depressive symptomatology in participants with a neurogenesis-centred biological susceptibility relative to those without such a susceptibility, we tested the interaction between various dietary/nutritional factors and neurogenesis-centred biological susceptibility status, using multivariable-adjusted logistic regression models. Specifically, we tested for interactions between: (i) dietary/nutritional factors (i.e., food consumption, nutrient intakes, and nutritional biomarker concentrations) and high versus low levels of %CC3-d on CD, (ii) dietary/nutritional factors and high versus low levels of %CC3-p on AD, (iii) dietary/nutritional factors and high versus low levels of %SOX2 on VoD, and (iv) dietary/nutritional factors and high versus low levels of %MAP2 on depressive symptomatology. HN readout classification was dichotomously determined by median split. Abbreviations: M, male; F, female; y, years; h, hours; CC3 cleaved caspase 3; SOX2, SRY (sex determining region Y)-box 2; MAP2, microtubule-associated protein 2. Image created using BioRender software. (A) adapted from our previously published schematics on the cohort and experimental design [9, 10].

As depicted in Figure 1A, the sample was derived from participants within the 3C cohort, a prospective cohort of older persons who provided repeated measures of cognitive function over 12 years [18]. We considered all dementia-free participants that had a baseline HN profile (*n* = 371), measured as part of a case–control study on CD status nested within the cohort [9, 13].

At baseline (1999–2000), face-to-face interviews were conducted to collect sociodemographic information, and fasting blood samples were collected to establish a plasma and serum biobank. Plasma samples were used to measure various nutritional biomarkers, whereas the serum samples were utilised to generate HN profiles for each participant using in vitro cellular HN assays [9, 10]. At the 2-year follow-up (2001–03), dietary habits and nutrient intakes were measured, and in-person neuropsychological assessments for CD, dementia, and depressive symptomatology were performed every two to three years over a 12-year period.

The 3C research protocol received approval from the Consultative Committee for the Protection of Persons participating in Biomedical Research at Kremlin-Bicetre University Hospital in Paris, France. All participants provided written informed consent. For further details, see Figure 1, footnote.

### Neurogenesis-centred biological susceptibility

A neurogenesis-centred biological susceptibility was differently defined for CD, dementia, and depressive symptomatology based on the HN measures associated with each outcome in our previous work [9, 10]. Briefly, serum samples collected at baseline (1999–2000) were used to generate neurogenesis profiles for each participant using in vitro HN cellular assays. We subsequently identified distinct associations between specific readouts of the neurogenic process and the three specified outcomes. These particular HN readouts were then used to establish susceptibility status (i.e., with/without altered HN) in our present analyses.

As depicted in Figure 1**,** each relevant HN marker was dichotomised by median split. Specifically, individuals with a neurogenesis-centred biological susceptibility to CD were categorised as those with higher levels of cell death during differentiation (i.e., %CC3-d ≥ 6%) [9]. Concerning dementia, individuals with a neurogenesis-centred biological susceptibility to Alzheimer’s disease (AD) had lower levels of cell death during *proliferation* (i.e., %CC3-p ≤ 1%), whereas individuals with a neurogenesis-centred biological susceptibility to vascular and other dementias (VoD) had lower levels of hippocampal progenitor cell integrity (i.e., %SOX2 ≤ 94%) [9]. Finally, individuals with a neurogenesis-centred biological susceptibility to later-life depressive symptomatology were defined as those with higher levels of hippocampal cell differentiation (i.e., %MAP2 ≥ 47%) [10].

### Dietary and nutritional factors

For present analyses, we used data on three aspects of diet/nutrition: (i) dietary habits/food consumption (available for *n* = 308 participants), (ii) nutrient intakes (*n* = 315), and (iii) nutrient biomarkers, including fatty acids (*n* = 279), vitamin D (*n* = 247), carotenoids (*n* = 255), and vitamin E (*n* = 255).

Briefly, at the 2-year follow-up visit (2001–02), dietary habits were assessed using the Food Frequency Questionnaire (FFQ), and nutrient intakes were ascertained through a 24-h dietary recall—all as previously described [19–21]. At baseline (1999–2000), the concentrations of 23 nutritional biomarkers (i.e., 12 fatty acids, 6 carotenoids, 25(OH)D, α- and γ-tocopherol, retinol, and transthyretin) were determined in total plasma as previously described [22–24]. See Table A.2 in the Supplementary Data for further details on the FFQ and dietary recall.

### Cognitive decline

Participants within our sample were classified as either cognitively stable or with accelerated CD based on performance in various cognitive tasks over 12 years, as previously described [13]. Participants with the worst slopes of decline were classified as those with accelerated CD, whereas participants with a CD below median value (i.e., >median slope) were classified as cognitively stable. See the Appendices in the Supplementary Data section for details.

### Dementia

All participants were assessed for dementia over the 12-year follow-up period. No participant within our sample had a dementia diagnosis at baseline. Clinical diagnosis of dementia was established and validated by an independent committee of neurologists, using the Diagnostic and Statistical Manual of Mental Disorders IV [25], as previously described [18].

For present analyses, dementia aetiology was considered as two main categories: (i) AD (i.e., all probable AD, possible AD and mixed dementia), and (ii) VoD (i.e., vascular dementia, Parkinson dementia, Lewy body dementia, and frontotemporal dementia). See the Appendices in the Supplementary Data section for details.

### Depressive symptomatology

Depressive symptomatology at baseline and throughout the 12-year follow-up was assessed using the validated Center for Epidemiologic Studies Depression (CES-D) scale [26]. Cases were defined as individuals with clinically relevant depressive symptoms, indicated by CES-D scores ≥17 in men and ≥23 in women [26–28], at any assessment over the follow-up period. See the Appendices in the Supplementary Data section for details.

### Covariates

Cardiometabolic risk factors were all assessed at baseline, including BMI (kg/m^2^), diabetes, hypertension, hypercholesterolemia, and fasting plasma levels of glucose, cholesterol, and triglycerides (measured by routine enzymatic methods). Medication use was also recorded. ApoE-ε4 genotype was defined as carrying at least one ε4 allele (relative to no ε4 allele), and lifestyle factors included regular physical activity, smoking status, and alcohol consumption. For further details on covariates, refer to Table 1**,** footnote.

**Table 1:** Baseline participant characteristics as stratified by outcome caseness (*n* = 371)

Measures	Cognitive Decline (CD)^a^			Incident dementia: Alzheimer's Disease (AD)^b^			Incident dementia: Vascular and other dementias (VoD)^c^			Depressive symptomatology^d^		
	Controls (*n* = 168)	Cases (*n* = 203)	Adjusted *P*^e^	Controls (*n* = 264)	Cases (*n* = 76)	Adjusted *P*^e^	Controls (*n* = 264)	Cases (*n* = 31)	Adjusted *P*^e^	Controls (*n* = 109)	Cases (*n* = 262)	Adjusted *P*^e^
Sociodemographic characteristics												
**Age (years)**	75 (4.1)	76 (4.5)	0.85	**75 (4.3)**	**77 (4.1)**	**0.008** ^**^	75 (4.3)	77 (4.7)	0.19	76 (4.3)	76 (4.4)	0.72
Sex; female	109 (65)	136 (67)	0.48	163 (62)	58 (76)	0.20	163 (62)	24 (80)	0.19	176 (67)	70 (63)	0.66
Education ≥ secondary school^f^	53 (32)	60 (30)	0.69	81 (31)	21 (28)	0.97	81 (31)	11 (37)	0.59	**190 (73)**	**71 (64)**	**0.054** ^**q**^
Health indicators												
BMI (kg m^−2^)	26.3 (3.72)	26.8 (4.41)	0.50	26.7 (4.14)	26.2 (4.72)	0.51	26.7 (4.12)	26.5 (2.91)	0.84	26.4 (4.13)	26.4 (3.81)	0.81
**Plasma total cholesterol (mmol L** ^**−1**^ **)**	5.19 (1.15)	5.38 (1.45)	0.15	5.80 (0.95)	5.93 (0.99)	0.26	**5.80 (0.95)**	**5.78 (0.86)**	**0.004** ^**^	5.8 (1.0)	5.9 (0.9)	0.59
**Plasma LDL cholesterol (mmol L** ^**−1**^ **)**	5.78 (0.91)	5.85 (0.98)	0.11	3.60 (0.83)	3.77 (0.88)	0.22	**3.60 (0.83)**	**3.65 (0.76)**	**0.003** ^**^	3.6 (0.8)	3.7 (0.8)	0.83
**Plasma HDL cholesterol (mmol L** ^**−1**^ **)**	3.59 (0.80)	3.68 (0.86)	0.49	1.60 (0.39)	1.50 (0.36)	0.35	**1.60 (0.39)**	**1.68 (0.37)**	**0.002** ^**^	1.6 (0.4)	1.6 (0.4)	0.77
Plasma triglycerides (mmol L^−1^)	1.60 (0.38)	1.57 (0.39)	0.97	1.32 (0.77)	1.29 (0.68)	0.75	1.32 (0.77)	1.40 (0.57)	0.90	1.3 (0.7)	1.4 (0.7)	0.21
**Plasma glucose (mmol L** ^**−1**^ **)**	1.28 (0.66)	1.36 (0.79)	0.27	5.26 (1.21)	5.34 (1.75)	0.89	**5.26 (1.21)**	**5.45 (1.11)**	**0.004** ^**^	**5.2 (1.1)**	**5.5 (1.7)**	**0.048** ^*^
Genetic risk factors												
**ApoE-ε4 carrier** ^g^	**19 (11)**	**52 (26)**	**<0.001** ^***^	**44 (17)**	**21 (28)**	**0.03** ^*^	44 (17)	5 (17)	0.33	50 (19)	22 (20)	0.55
Medical factors												
Hypertension^h^	124 (74)	160 (79)	0.86	198 (75)	57 (75)	0.49	198 (75)	28 (93)	0.11	202 (77)	83 (75)	0.58
**Diabetes** ^i^	**10 (6)**	**26 (13)**	**0.03** ^*^	20 (8)	9 (12)	0.54	**20 (8)**	**7 (25)**	**0.02** ^*^	10 (26)	11 (10)	0.54
**Hypercholesterolemia** ^j^	101 (60)	122 (60)	0.45	147 (56)	49 (64)	0.61	**147 (56)**	**25 (83)**	**0.01** ^*^	152 (58)	73 (66)	0.15
Antecedents of CVD^k^	44 (26)	68 (33)	0.81	75 (29)	23 (30)	0.43	75 (29)	14 (47)	0.12	86 (33)	28 (25)	0.09
Medication												
**Antihypertensive medication** **use** ^l^	**87 (52)**	**129 (64)**	**0.02** ^*^	151 (57)	44 (58)	0.36	**151 (57)**	**21 (70)**	**0.02** ^*^	149 (57)	69 (62)	0.21
**Diabetic medication use** ^m^	13 (8)	14 (7)	0.66	19 (7)	3 (4)	0.29	**19 (7)**	**5 (17)**	**0.056** ^**q**^	17 (7)	10 (9)	0.44
Lipid lowering medication use^n^	52 (31)	78 (38)	0.11	88 (34)	32 (42)	0.46	88 (34)	10 (33)	0.23	88 (34)	39 (35)	0.53
**Psychotropics and antidepressants use** ^o^	41 (24)	68 (33)	0.19	**68 (26)**	**27 (36)**	**0.09** ^**q**^	**68 (26)**	**13 (43)**	**0.03** ^*^	75 (29)	36 (32)	0.68
Vitamin D supplement use	5 (3)	10 (5)	0.96	12 (5)	0 (0)	0.99	12 (5)	3 (10)	0.11	9 (3)	6 (5)	0.15
Lifestyle characteristics												
Regular physical exercise^p^	55 (37)	45 (28)	0.25	78 (34)	16 (29)	0.62	78 (34)	4 (20)	0.19	65 (25)	30 (27)	0.39
Alcohol use (glasses per week)	15 (18)	13 (14)	0.26	15 (17)	10 (11)	0.22	15 (17)	14 (15)	0.41	14 (17)	13 (15)	0.44
Smoking status			0.43			0.42			0.82			0.30
Never	107 (64)	139 (68)	–	167 (63)	57 (75)	–	167 (63)	20 (67)	–	169 (65)	77 (69)	–
Former	52 (31)	54 (27)	–	83 (32)	15 (20)	–	83 (32)	8 (27)	–	77 (29)	31 (28)	–
Current	9 (5)	10 (5)	–	13 (5)	4 (5)	–	13 (5)	2 (7)	–	16 (6)	3 (3)	–

Values represent mean (SD) or *N* (%) of non-missing values. Characteristics (and associated values) in bold are covariates, all of which are controlled for in relevant models. Abbreviations: ApoE-ε4, allele ε4 for the apolipoprotein E gene; HDL, high-density lipoprotein; LDL, low-density lipoprotein; BMI, body mass index; CVD, cardiovascular disease.

^a^Participants were classified as either cognitively stable or with accelerated CD based on their average performance in five neuropsychological tests, i.e., the Mini-Mental State Examination, the Benton Visual Retention Test, the Isaac’s Set Test, and the Trail-Making Test parts A and B across five follow-up visits across the 12-year study duration [13].

^b^AD cases included all diagnoses of probable AD, possible AD, and mixed dementia and was established and validated by an independent committee of neurologists.

^**c**^VoD included all diagnoses of vascular dementia, Parkinson dementia, Lewy body dementia, and frontotemporal dementia and was established and validated by an independent committee of neurologists.

^**d**^Assessed using the Center for Epidemiological Studies-Depression (CES-D) scale [26, 77]. CES-D scores ≥17 in men and ≥23 in women were used as indicators of a high depressive symptomatology.

^**e**^Estimated using logistic regressions controlling for age, sex, and education. FDR correction was applied to control for multiple testing; alpha threshold 0.05; *P* values represent FDR-corrected *P* values.

^**f**^Education was based on the highest level of attainment and considered dichotomously: either as no or primary level education only or as secondary/high school level and above.

^**g**^ApoE genotype was considered dichotomously: presence of at least one ε4 allele.

^**h**^Blood pressure ≥ 140/90 mmHg or antihypertensive medication use.

^**i**^Glucose ≥7.2 mmol/L or antidiabetic medication use.

^**j**^Fasting plasma total cholesterol ≥6.2 mmol/L or lipid-lowering medication use.

^**k**^History of cardiovascular or cerebrovascular disease**.**

^**l**^Includes all antihypertensive drugs, calcium channel blockers, diuretics, beta-blockers, and drugs acting on the renin-angiotensin system.

^**m**^Includes all antidiabetic drugs except insulin**.**

^**n**^Includes all statins, fibrates, or bile acid sequestrants.

^**o**^Includes all psycholeptics and psychoanaleptics—antidepressants, psychostimulants, and nootropics.

^**p**^Practice and intensity of physical exercise was assessed using a physical activity questionnaire for older adults [78]. Regular exercise was classified as doing sport regularly or having at least 1h of leisure or household activity per day, as described in detail in [79].

^**q**^Also adjusted for in further analyses where relevant.

^*^ *P* < 0.05.

^**^ *P* < 0.01.

^***^ *P* < 0.001.

### Statistical analysis

Data analyses were conducted using R (v.4.3.1 [29]). Baseline characteristics of the sample were expressed as means and standard deviations (SD) for continuous variables and frequencies and percentages for categorical variables. Baseline characteristic comparisons were tested using logistic regression models for the three specified outcomes.

As depicted in Figure 1B, using logistic regression models for CD, dementia, and depressive symptomatology, interaction analyses were performed to examine whether dietary/nutritional factors (i.e., dietary habits/food consumption from the FFQ, nutrient intakes from 24-h dietary recall, and nutrient biomarkers, including plasma concentrations of fatty acids, vitamin D, carotenoids, and vitamin E) could modify the increased risk associated with a neurogenesis-centred biological susceptibility and future CD, dementia (AD and VoD), and depressive symptomatology. Regression models were primarily adjusted for age, sex, education, age of dementia onset (AD and VoD only), or baseline depressive symptoms (depression only) (Model 1), followed by further adjustment for physical exercise and other covariates (Model 2).

The same strategy of selecting covariates was applied to all models. Briefly, we adjusted factors that were significantly different at *P* < 0.05 between cases and controls for the outcomes of interest (CD, dementia, and depressive symptomatology) using logistic regression analyses controlled for age, sex, and education. All covariates are bolded in Table 1. Although not significantly different at *P* < 0.05, physical activity, a potential confounder for all outcomes, was also introduced in Model 2 for all analyses.

For all models, false discovery rate (FDR) correction, with a threshold of *P* < 0.05, was applied to account for multiple testing. Further details on the proposed models can be found in the Appendices in the Supplementary Data section.

When significant interactions between dietary/nutritional factors and neurogenesis-centred biological susceptibility status on CD/dementia/depressive symptomatology were observed, stratification analyses were subsequently performed using multivariable-adjusted logistic regression models for each subgroup (i.e., individuals with or without a neurogenesis-centred biological susceptibility), as described above.

## Results

### Sample characteristics

The characteristics of our sample by CD, dementia status, and depressive symptomatology are detailed in Table 1. Participant cognition was assessed for a mean of 8.5 years, and age of dementia onset was 85 years on average. For depressive symptomatology, 29% reported high depressive symptomatology throughout the 12-year study period.

Participants that later developed AD and VoD were more likely to have a neurogenesis-centred biological susceptibility at baseline (AD: controls: *n* = 135 (52%) vs. cases: *n* = 49 (64%), *P* = 0.047; VoD: controls: *n* = 125 (48%) vs. cases: *n* = 22 (73%); *P* = 0.006). However, no significant differences in neurogenesis-centred biological susceptibility status were observed between cases and controls for CD and depressive symptomatology (CD: controls: *n* = 87 (52%) vs. cases: *n* = 112 (55%), *P* = 0.12; depressive symptoms: controls: *n* = 124 (48%) vs. cases: *n* = 59 (53%); *P* = 0.35).

For comparisons of the participant characteristics between the whole sample and the various subsamples (i.e., dietary/nutritional factors excluding missing data) used to inform present analyses, see Table A.1 available in the Appendices in the Supplementary Data section. No differences in the characteristics between the whole sample and the various subsamples were observed.

To determine whether diet/nutrition could modify the risk associated with altered HN and future CD, dementia (AD and VoD), and depressive symptomatology, we first tested the interaction between various dietary/nutritional factors and neurogenesis-centred biological susceptibility status on these outcomes (Table A.2 available in the Appendices in the Supplementary Data section).

### Diet does not modify the increased risk associated with altered HN and future CD but does modify the risk associated with future dementia

As shown in Table A.2 (available in the Appendices in the Supplementary Data section), we found no significant interactions between having a neurogenesis-centred biological susceptibility (as defined by higher cell death during differentiation levels [%CC3-d ≥ median]) and any dietary or nutritional factor on CD.

However, we did find that the increased risk associated with having a neurogenesis-centred biological susceptibility on future dementia outcomes were modified by diet/nutrition. Specifically, we found significant interactions between having a neurogenesis-centred biological susceptibility (as defined by lower cell death during proliferation levels [%CC3-p ≤ median]) and plasma lycopene concentrations (FDR-adjusted *P* = 0.01), red meat consumption (FDR-adjusted *P* = 0.008), and poultry consumption (FDR-adjusted *P* = 0.02) on AD risk. Moreover, we found a significant interaction between having a neurogenesis-centred biological susceptibility (as defined by lower hippocampal progenitor cell integrity levels [%SOX2 ≤ median]) and vitamin D consumption on VoD risk (FDR-adjusted *P* = 0.04).

#### Increased plasma lycopene levels, red meat consumption, and reduced poultry consumption are all associated with an increased risk for future AD but only in individuals with a neurogenesis-centred biological susceptibility

**Figure 2:**
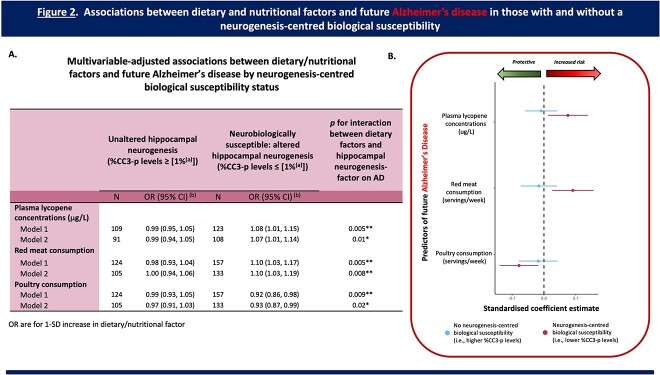
**Associations between dietary/nutritional factors and future AD in those with and without a neurogenesis-centred biological susceptibility 12 years prior to condition onset.** (A) Table presenting the multivariable-adjusted associations between dietary/nutritional factors and future AD by neurogenesis-centred biological susceptibility status (i.e., %CC3-p levels—cell death during proliferation). (a) Represents the median for %CC3-p levels. (b) OR are for 1-SD increase in dietary factor. Analysis: Logistic Regression. Model 1: adjusted for age, sex, level of education, and age of dementia onset. Model 2: fully adjusted. Adjusted as per Model 1 plus physical exercise, APOe4 carrier status, and psychotropic medication use. (B) Regression coefficient plot representing the associations between key dietary/nutritional factors and future AD in those with and without a neurogenesis-centred biological susceptibility. Plasma lycopene concentrations (a carotenoid biomarker) and consumption profiles (red meat and poultry) all significantly modified the risk for future AD, but only in individuals with a neurogenesis-centred biological susceptibility (i.e., lower (≤median) baseline levels of cell death during proliferation). Abbreviations: CC3, Cleaved Caspase 3; OR, odds ratio; CI, confidence intervals; ApoE-ε4, allele ε4 for the apolipoprotein E gene. FDR corrected *P* values; ^*^P < 0.05; ^**^P < 0.01.

As depicted in Figure 2, amongst individuals with a neurogenesis-centred biological susceptibility (%CC3-p), higher plasma lycopene concentrations were associated with an increased risk of AD (OR [95% CI] = 1.07 [1.01, 1.14], *P* (uncorrected) = 0.04), whereas there was no significant association amongst those without this neurogenesis-centred biological susceptibility (OR [95% CI] = 0.99 [0.94, 1.05, *P* (uncorrected) = 0.83).

With respect to red meat and poultry consumption; amongst individuals with this same neurogenesis-centred biological susceptibility, higher red meat consumption (OR [95% CI] = 1.10 [1.03, 1.19], *P* (uncorrected) = 0.008) and reduced poultry consumption (OR [95% CI] = 0.93 [0.87, 0.99], *P* (uncorrected) = 0.03) were both associated with an increased risk of AD, whereas there were no significant associations amongst those without this susceptibility (meat: OR [95% CI] = 1.00 [0.94, 1.06], *P* (uncorrected) = 0.95; poultry: OR [95% CI] = 0.97 [0.91, 1.03], *P* (uncorrected) = 0.38).

#### Increased vitamin D consumption is associated with an increased risk for future VoD but only in individuals with a neurogenesis-centred biological susceptibility

**Figure 3:**
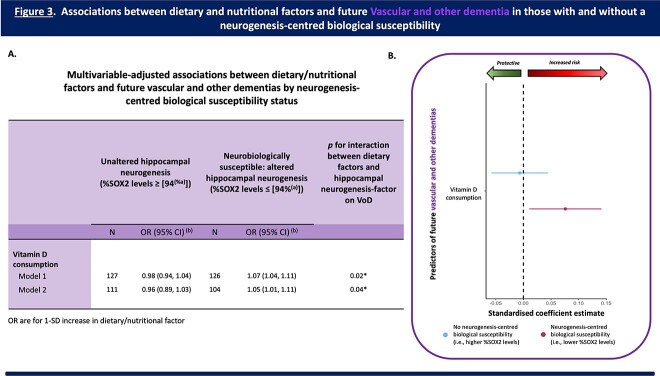
**Associations between dietary/nutritional factors and future VoD in those with and without a neurogenesis-centred biological susceptibility 12 years prior to condition onset.** (A) Table presenting the multivariable-adjusted associations between dietary/nutritional factors and future VoD by neurogenesis-centred biological susceptibility status (i.e., %SOX2 levels—hippocampal progenitor cell integrity). (a) Represents the median for %SOX2 levels. (b) OR are for 1-SD increase in dietary factor. Analysis: Logistic Regression. Model 1: adjusted for age, sex, level of education, and age of dementia onset. Model 2: fully adjusted. Adjusted as per Model 1 plus physical exercise, plasma cholesterol levels, plasma glucose levels, diabetes, hypercholesterolemia, antihypertensive medication use, diabetic medication use, and psychotropic medication use. (B) Regression coefficient plot representing the associations between key dietary/nutritional factors and future VoD in those with and without a neurogenesis-centred biological susceptibility. Increased vitamin D consumption increased the risk for future VoD, but only in individuals with a neurogenesis-centred biological susceptibility (i.e., reduced (≤median) baseline levels of hippocampal progenitor cell integrity). Abbreviations: SOX2, SRY (sex determining region Y)-box 2; OR, odds ratio, CI, confidence intervals, ApoE-ε4, allele ε4 for the apolipoprotein E gene. FDR corrected *P* values; ^*^P < 0.05.

Figure 3 shows that amongst individuals with a neurogenesis-centred biological susceptibility (%SOX2), higher vitamin D consumption was associated with an increased risk of VoD (OR [95% CI] = 1.05 [1.01, 1.11], *P* (uncorrected) = 0.04), whereas there was no significant association amongst those without this neurogenesis-centred biological susceptibility (OR [95% CI] = 0.96 [0.89, 1.03, *P* (uncorrected) = 0.26).

### Diet modifies the association between altered HN and increased late-life depressive symptomatology

In addition to finding that diet/nutrition could modify the increased risk associated with altered HN and dementia, we found a significant interaction between having a neurogenesis-centred biological susceptibility (as defined by higher hippocampal cell differentiation levels [%MAP2 ≥ median]) and plasma γ-tocopherol concentrations on later-life depressive symptomatology (FDR-adjusted *P* = 0.03).

#### Increased plasma γ-tocopherol levels are associated with increased depressive symptomatology but only in individuals with a neurogenesis-centred biological susceptibility

**Figure 4:**
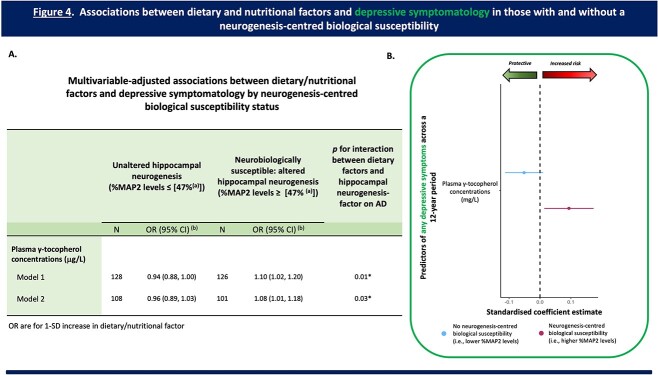
**Associations between dietary/nutritional factors and depressive symptomatology across a 12-year period in those with and without a neurogenesis-centred biological susceptibility. **(A) Table presenting the multivariable-adjusted associations between dietary/nutritional factors and any depressive symptomatology across a 12-year period by neurogenesis-centred biological susceptibility status (i.e., %MAP2 levels—hippocampal cell differentiation). (a) Represents the median for %MAP2 levels. (b) OR are for 1-SD increase in dietary factor. Analysis: logistic regression. Model 1: adjusted for age, sex, level of education, and baseline depressive symptomatology. Model 2: fully adjusted. Adjusted as per Model 1 plus physical exercise, and plasma glucose levels. (B) Regression coefficient plot representing the associations between key dietary factors and any depressive symptomatology in those with and without a neurogenesis-centred biological susceptibility. Increased plasma γ-tocopherol concentrations (a vitamin E biomarker) increased the risk of having any depressive symptomatology, but only in individuals with a neurogenesis-centred biological susceptibility (i.e., higher (≥median) baseline levels of hippocampal cell differentiation). Abbreviations: MAP2, microtubule-associated protein 2; OR, odds ratio; CI, confidence intervals. FDR corrected *P* values; ^*^P < 0.05.

As shown in Figure 4, amongst individuals with a neurogenesis-centred biological susceptibility (%MAP2), higher plasma γ-tocopherol concentrations were associated with an increased incidence of later-life depressive symptoms (OR [95% CI] = 1.08 [1.01, 1.18], *P* (uncorrected) = 0.048), whereas there was no significant association amongst those without this neurogenesis-centred biological susceptibility (OR [95% CI] = 0.96 [0.89, 1.03, *P* (uncorrected) = 0.22).

## Discussion

Diet has been identified as one modifiable factor that may foster healthier ageing [30–33] and, here, we promote the significance of diet as a central element in shaping the trajectory of healthy brain ageing. Specifically, our findings highlight how dietary habits and nutritional factors can interact with altered neurogenesis to modify risk for future dementia and late-life depressive symptoms (Figure A.1 available in the Appendices in the Supplementary Data section).

In the context of AD, our research uncovered several noteworthy findings. We observed that reduced red meat consumption and increased poultry consumption may offer protection against AD but only in individuals with a neurogenesis-centred biological susceptibility. These findings align with previous studies focusing on these specific food groups [34–36] and the Mediterranean diet [14, 15, 37], which promotes moderate to high intake of fish and poultry, along with limited consumption of red meat for optimal ageing [38–40].

However, whilst greater adherence to the Mediterranean diet has been shown to reduce the risk for AD and is considered to positively influence the nine hallmark features of ageing [41], it is important to note that we did not observe a significant interaction between Mediterranean diet scores and HN on AD in our study. This could indicate that specific food groups rather than overall dietary patterns may have a stronger impact on the neurogenic process. Moreover, a systematic review recently reported limited and inconsistent evidence around associations between adherence to the Mediterranean diet and cerebral vascular-related biomarkers (i.e., hippocampal volume and white matter intensity), highlighting an important gap in the literature and the need for further research specifically on dietary patterns and brain ageing [42].

The observed associations in meat and poultry consumption in individuals with a neurogenesis-centred biological susceptibility and increased future AD could, however, be attributed to the impact of high-fat (red meat) and low-fat (poultry) protein sources on the body and brain. Protein, particularly amino acid metabolism, plays a crucial role in maintaining the integrity of neuronal membranes, and regulating adult neurogenesis [43]. It also aids in muscle strength and retention in ageing adults—factors that may be important for dementia prevention [5, 44, 45].

Importantly, an excess of high-fat protein intake, such as red meat, increases the risk of cardiovascular diseases and diabetes [46], an outcome not observed for poultry consumption [47]. This may explain why high consumption of red meat, in particular, is not associated with reduced dementia risk [48]. Additionally, preclinical studies indicate that diets high in saturated fat can heighten oxidative stress, neuroinflammation, and altered HN [49–52]—changes all associated with dementia [5, 53, 54]. Moreover, cardiovascular disease and diabetes can significantly impair the neurogenic process [55–57]. Thus, in individuals with a neurogenesis-centred biological susceptibility, increased consumption of high-fat protein sources (red meat) might exacerbate an already compromised biological system and/or impact other key biological systems (i.e., cardiovascular system), accelerating the progression to AD. Conversely, increased consumption of low-fat protein sources (poultry) may help to buffer the HN-associated alterations. However, more work is needed to substantiate this.

Contrary to some previous studies [58, 59], we also found a positive association between plasma lycopene concentrations—a carotenoid biomarker—and AD risk, but only in individuals with a neurogenesis-centred biological susceptibility. However, overall, there is insufficient evidence to draw firm conclusions or tease apart direct effects of lycopene on future dementia risk [60], so it is presently unclear what this finding represents in the wider context of AD.

One potential explanation is that this could be a compensatory response in an attempt to mitigate the impact of any HN-associated impairments. Compensatory responses have been previously reported in the context of AD, where an increase in serum/plasma levels of glutamine (a neuroprotectant) and BDNF (a key regulator of neuronal growth and survival) appear early, followed by a decline in the advanced stages of the condition [61, 62].

In the context of our work, preclinical studies have consistently reported on the neuroprotective effects of lycopene by alleviating oxidative stress and suppressing production of inflammatory cytokines [63], and have shown how lycopene can specifically reduce neural stem cell death [64, 65]. Therefore, for individuals with this neurobiological susceptibility, increased lycopene could potentially signify a biological compensatory mechanism, aimed at rectifying or mitigating the impact of earlier neurobiological changes associated with AD progression.

Furthermore, it is noteworthy to emphasise that in our earlier research [9], we hypothesised that a decrease in hippocampal cell death during proliferation (i.e., a neurogenesis-centred susceptibility for AD) could signify an overall increase in the neurogenic process, which is also contrary to what is observed in AD post-mortem studies [5]. However, we and others share the belief that an early surge in HN in the trajectory of AD in itself serves as a compensatory neurobiological response [66]. Therefore, the observed elevation in lycopene concentrations may be closely linked to the concurrently observed compensatory increase in HN. However, future research is needed to substantiate this and should seek to understand how lycopene concentrations evolve across the trajectory of AD, particularly in individuals neurobiologically susceptible.

Surprisingly, we also observed a positive association between (i) vitamin D consumption and future VoD, and (ii) plasma concentrations of γ-tocopherol (a vitamin E biomarker) and depressive symptomatology—but again only in individuals with a neurogenesis-centred biological susceptibility. Generally, these findings do not align with other research, including work from the wider 3C cohort, which supports a protective association between vitamin D and dementia [24, 67–69], and vitamin E and depression [70, 71]. Moreover, whilst HN in the context of VoD and depression has been relatively understudied, our previously reported HN findings were not counterintuitive [9, 10]. Thus, we are unable to draw definitive conclusions for these findings.

However, it is important to emphasise that here we are presenting a cross-sectional snapshot of how HN and diet interact 10–12 years prior to condition onset. Therefore, we speculate that these findings could also potentially signify early compensatory responses to having a neurogenesis-centred susceptibility, given that antioxidant vitamins (such as vitamins D and E) are key regulatory factors of neurogenesis [72–74]. To our knowledge, compensatory biological responses (outside of stress/(neuro)inflammation) in the context of depression and VoD have been largely unexplored. Therefore, further research is needed to substantiate our speculations or determine whether these dietary and nutritional outcomes are influencing other downstream processes that subsequently promote VoD pathology and depressive symptomatology years later.

Our study strengths include the use of a well-characterised prospective cohort to evaluate the interaction between dietary/nutritional factors and neurogenesis-centred susceptibility status on CD, dementia, and depressive symptomatology. This study is also the first to classify neurobiological susceptibility to these conditions using HN profiles 12 years prior to onset.

However, there are limitations. As with any observational study, associations between nutritional biomarkers and our outcomes may be influenced by residual confounding. Furthermore, other unmeasured biomarkers might play an important role (e.g., flavonoids [75, 76]). Additionally, dietary intake data primarily captures short-term exposure and are susceptible to measurement errors, and it is important to note that the nutritional and dietary assessments were performed at different times with a 2-year interval. Moreover, lifelong dietary habits are also important, and our study only provides a snapshot. To understand the impact of diet/nutrition more fully across the trajectory of dementia and LLD in individuals with a neurobiological susceptibility, it would be profitable for future research to explore the dynamic interaction between diet and HN at multiple timepoints. Finally, given the exploratory nature of our study, it is difficult to conclude on the generalisability of our findings.

In conclusion, our research highlights the critical role for diet/nutrition in modifying the risk for future dementia and depressive symptomatology specifically in individuals with a neurogenesis-centred biological susceptibility. Our work highlights the importance of understanding the factors that promote or hinder brain health in subgroups of older individuals.

## Supplementary Material

aa-24-0233-File002_afae042
