# Supplementary material for: Association of dietary and nutritional factors with cognitive decline, dementia, and depressive symptomatology in older individuals according to a neurogenesis-centred biological susceptibility to brain ageing
Source: Age Ageing. 2024 May 15;53(Suppl 2):ii47–59. doi: 10.1093/ageing/afae042 (PMC11094407; doi:10.1093/ageing/afae042)
Supplement: aa-24-0233-File002_afae042 [file aa-24-0233-file002_afae042.docx]

**Association of dietary and nutritional factors with cognitive decline, dementia, and depressive symptomatology in older individuals according to a neurogenesis-centred biological susceptibility to brain ageing**

**Andrea Du Preez^1^, Sophie Lefèvre-Arbogast^2^, Raúl González-Domínguez^3,4^, Vikki Houghton^1^, Chiara de Lucia^1^, Hyunah Lee^1^, Dorrain Y Low^5^, Catherine Helmer^2^, Catherine Féart^2^, Cécile Delcourt^2^, Cécile Proust-Lima^2^, Mercè Pallàs^6^, Alex Sánchez-Pla^3,4^, Mireia Urpi-Sardà^3,4^, Silvie R. Ruigrok^7^, Barbara Altendorfer^8^, Ludwig Aigner^8^, Paul J Lucassen^7^, Aniko Korosi^7^, Claudine Manach^5^, Cristina Andres-Lacueva^3,4^, Cécilia Samieri^2^, Sandrine Thuret^1, 9^**

^1^ Department of Basic and Clinical Neuroscience, Maurice Wohl Clinical Neuroscience Institute, Institute of Psychiatry, Psychology and Neuroscience, King’s College London, London SE5 9NU, UK; ^2^ University of Bordeaux, Inserm, Bordeaux Population Health Research Center, UMR 1219, F-33000 Bordeaux, France; ^3^ Nutrition, Food Science and Gastronomy Department, Faculty of Pharmacy and Food Science, University of Barcelona, 08028 Barcelona, Spain; ^4^ CIBER Fragilidad y Envejecimiento Saludable (CIBERFES), Instituto de Salud Carlos III,0828, Barcelona; ^5^ Université Clermont Auvergne, INRA, UMR1019, Human Nutrition Unit, F-63000 Clermont Ferrand, France; ^6^ Pharmacology Section, Department of Pharmacology, Toxicology and Medicinal Chemistry, Faculty of Pharmacy and Food Sciences, and Institute of Neurosciences, University of Barcelona, Av. Joan XXIII, 27-31, E-08028, Barcelona, Spain; ^7^ Brain Plasticity Group, Swammerdam Institute for Life Sciences, Center for Neuroscience, University of Amsterdam, 1098 XH Amsterdam, The Netherlands; ^8^ Institute of Molecular Regenerative Medicine, Spinal Cord Injury and Tissue Regeneration Center Salzburg, Paracelsus Medical University, Salzburg 5020, Austria; ^9^ Department of Neurology, University Hospital Carl Gustav Carus, Technische Universität Dresden, 01307 Dresden, Germany.

**SUPPLEMENTARY METHODS AND DATA**

**CONTENT**

- **Methods and materials**
  - Dietary habits and nutrient intake
  - Cognitive decline
  - Dementia
  - Depressive symptomatology
  - Statistical analysis
- **Results Tables**
  - Table A.1. Baseline characteristics of the whole sample and the various dietary and nutritional factor subsamples (excluding missing data).
  - Table A.2. Interaction analyses between dietary and nutritional factors and neurogenesis-centred biological susceptibility status on cognitive decline, dementia, and depressive symptomatology using multivariable-adjusted logistic regression models.
- **Figure A.1.** Schematic overview of key risk (+) and protective (-) dietary and nutritional factors for dementia and depressive symptomatology in individuals with a neurogenesis-centred biological susceptibility.
- **References**

**METHODS AND MATERIALS**

**Dietary habits and nutrient intake**

At the two-year follow-up visit (2001-2002), dietary habits were assessed using the Food Frequency Questionnaire (FFQ) to measure the frequency of consumption (in servings per week) of 19 food/beverage categories, as previously described [1,2]. Additionally, nutrient intakes were ascertained through a 24h dietary recall administered concomitantly to the FFQ. The 24h recall consisted of reporting all meals and beverages consumed during a 24h period. Individual daily intakes in macro- and micronutrients were estimated by multiplying food/beverage intakes by their nutrient content using standard nutrient composition databases – all as previously described [3].

Additionally, using data from the FFQ, a Mediterranean diet score was generated by adding the scores for each food group considered to be part of the Mediterranean diet as previously described [4]. Briefly, food items were converted into number of servings per week and then aggregated into 20 food and beverage groups. Food groups considered to be part of the Mediterranean diet (i.e., vegetables, fruits, legumes, cereals [including bread], pasta and rice [whole and refined grains], fish, meat, dairy products, and alcohol) were subsequently identified and the number of servings per week for each food group was determined. A Mediterranean diet score was then computed whereby a value of 0 or 1 was assigned to each food group using sex-specific medians of the population as cut-offs. A total score was generated by adding the scores (0 or 1 point) for each food category for each participant. Thus, the score could range from 0 to 9, with higher scores indicating greater adherence to the Mediterranean diet.

**Cognitive decline**

Participants within our sample were classified as either cognitively stable or with accelerated CD as previously described [5]. Briefly, using linear mixed models, individual slopes of cognitive change for each participant over the 12-year follow-up period were estimated. The primary outcome represented the change in a composite score of global cognition, which was defined as the average of Z-scores of five neuropsychological tests: (i) the Mini-Mental State Examination (assessing global cognitive performance)[6], (ii) the Benton Visual Retention Test (assessing visual working memory and attention)[7], (iii) the Isaac’s Set Test (assessing verbal fluency)[8], (iv) the Trail-Making Test part A (assessing processing speed)[9], and (v) the Trail-Making Test part B (assessing executive functioning)[9], across the five follow-up visits. Participants with the worst slopes of decline were classified as those with accelerated CD, whereas participants with a CD below median value (i.e., >median slope) were classified as cognitively stable.

**Dementia**

In addition to measures of cognition, all participants were assessed for dementia across the 12-year follow-up period. No participant within our sample had a dementia diagnosis at baseline. Clinical diagnosis of dementia was established and validated by an independent committee of neurologists, using the Diagnostic and Statistical Manual of Mental Disorders IV[10], as previously described [11]. Briefly, the final diagnosis of dementia and subtype was made based on all available information, including data on cognitive functioning and daily activities, severity of cognitive disorders (i.e., Clinical Dementia Rating Scale), and, where possible, hospitalization records, computerized tomography (CT) scans and magnetic resonance images [11], and functional assessment (i.e., assessment of disabilities using the Katz Index of Activities of Daily Living [12], the Lawton Instrumental Activities of Daily Living Scale [13], and the Rosow and Breslau scales [11,14].

Dementia subtyping was based on the National Institute of Neurological and Communicative Disorders and Stroke–Alzheimer’s Disease and Related Disorders Association criteria for Alzheimer’s Disease (AD), and on the National Institute of Neurological Disorders and Stroke–Association Internationale pour la Recherche et l’Enseignement en Neurosciences criteria for vascular dementia [15,16]. Mixed dementia was defined as diagnosis of AD with either cerebrovascular lesions on brain imaging or a documented history of stroke and presence of prominent executive function deficits in addition to an AD-type cognitive profile. Seven dementia subtypes were identified but due to sample size limitations subtypes were pooled together into two main categories for present analyses: (1) AD (i.e., all probable AD, possible AD, and mixed dementia) and (2) vascular and other dementias (VoD, i.e., vascular dementia, Parkinson dementia, lewy body dementia and frontotemporal dementia).

**Depressive symptomatology**

Depressive symptomatology at baseline and throughout the 12-year follow-up was evaluated by a trained psychologist using the validated Center for Epidemiologic Studies Depression (CES-D) scale [17], which is also a valid and reliable measure of depressive symptomatology in older adult populations [18,19]. The CES-D is a 20-item self-report questionnaire designed to evaluate the frequency of depressive symptoms experienced over the past week. Each item is scored from 0 (rarely) to 3 (most of the time), with a total score ranging from 0 to 60 and increasing with the level of severity of depressive symptomatology. As previously validated in a French population, scores of ≥17 in men and of ≥23 in women were used as indicators of a clinically relevant level of depressive symptomatology [17,20,21]. Cases represented individuals with depressive symptoms at any assessment over the study duration. When the CES-D scale was not fully completed, the interviewers mentioned if it was because of severe depression. When more than four items out of 20 were missing because of severe depression as ascertained by the psychologist, the participant was considered as having high depressive symptoms. Caseness for depressive symptomology was not based on antidepressant use, e.g., participants with no depressive symptoms but taking psychoactive medication (including antidepressants) were classified as controls. However, only 30% of all participants, irrespective of symptomology, were taking medication during the study. Antidepressant use was however considered in all analyses.

**Statistical analysis**

Interaction analyses were performed to examine whether dietary/nutritional factors could modify the increased risk associated with a neurogenesis-centred biological susceptibility and future CD, dementia (AD and VoD), and depressive symptomatology. Specifically, to study the interactions between the various dietary/nutritional factors and neurogenesis-centred biological susceptibility (i.e., %CC3-differentiation) status on future CD, logistic regression models primarily adjusted for age, sex, and education were performed (Model 1), followed by further adjustment of physical activity, APOe4 carrier status, diabetes, and antihypertensive medication use (Model 2).

To test interactions between the various dietary/nutritional factors and neurogenesis-centred biological susceptibility (i.e., %CC3-proliferation) status on future AD, logistic regression models primarily adjusted for age, sex, education, and age of dementia onset were performed (Model 1), followed by further adjustment of physical activity, APOe4 carrier status, and psychotropic medication use (Model 2).

Interactions between the various dietary/nutritional factors and neurogenesis-centred biological susceptibility (i.e., %SOX2) status on future VoD were also tested using logistic regression models primarily adjusted for age, sex, education, and age of dementia onset (Model 1), followed by further adjustment of physical activity, plasma cholesterol and glucose levels, diabetes, hypercholesterolemia, and antihypertensive, diabetic, and psychotropic medication use (Model 2).

Finally, to study the interactions between the various dietary/nutritional factors and neurogenesis-centred biological susceptibility (i.e., %MAP2) status on incident depressive symptomatology, logistic regression models primarily adjusted for age, sex, education, and baseline depressive symptomatology were performed (Model 1), followed by further adjustment of physical activity, and plasma glucose concentrations (Model 2).

Where significant interactions between dietary/nutritional factors and neurogenesis-centred biological susceptibility status on CD/dementia/depressive symptomatology were observed, stratification analyses were subsequently performed using multivariable-adjusted logistic regression models for each subgroup (i.e., individuals with or without a neurogenesis-centred biological susceptibility) as detailed above.

| **Table A.1. Baseline characteristics of the whole sample and the various dietary and nutritional factor subsamples (excluding missing data)** | | | | | | |
| --- | --- | --- | --- | --- | --- | --- |
| **Measures** | **Whole sample**  **(n=371)** | **Fatty acid biomarker subsample**  **(n=279)** | **Transthyretin and Vitamin D biomarker subsample**  **(n=247)** | **Carotenoid and Vitamin E biomarker subsample**  **(n=255)** | **Food frequency questionnaire data subsample**  **(n=308)** | **Dietary recall data subsample**  **(n=315)** |
| **Neurogenesis-centred biological susceptibility as defined by hippocampal neurogenesis profiles at baseline *^(a)^*** | | | | | | |
| Low levels of hippocampal progenitor cell integrity (i.e., %SOX2 levels below median cut-off) | 184 (50) | 129 (46) | 125 (51) | 124 (49) | 157 (51) | 159 (51) |
| Low levels of cell death during proliferation (i.e., %CC3 levels below median cut-off) | 199 (51) | 146 (52) | 132 (53) | 131 (51) | 170 (55) | 172 (55) |
| High levels of cell death during differentiation (i.e., %CC3 levels above median cut-off) | 185 (50) | 124 (44) | 120 (49) | 125 (49) | 153 (50) | 156 (50) |
| High levels of neuronal differentiation  (i.e., %MAP2 levels above median cut-off) | 184 (50) | 131 (47) | 122 (50) | 126 (49) | 154 (50) | 156 (50) |
| **Neuropsychiatric/pathological outcomes** | | | | | | |
| Cognitive decline *^(b)^* | 203 (55) | 151 (54) | 117 (47) | 123 (48) | 159 (52) | 162 (51) |
| Alzheimer’s disease *^(c)^* | 76 (21) | 58 (21) | 69 (31) | 55 (22) | 60 (20) | 61 (19) |
| Vascular and other dementias *^(d)^* | 30 (8) | 19 (7) | 26 (15) | 23 (9) | 26 (8) | 27 (9) |
| Depressive symptomatology *^(e)^* | 111 (30) | 85 (31) | 68 (28) | 71 (28) | 90 (29) | 91 (29) |
| **Sociodemographic characteristics** | | | | | | |
| Age (years) | 76 (4.3) | 76 (4.3) | 75 (4.4) | 76 (4.3) | 75 (4.3) | 75 (4.3) |
| Sex; female | 245 (66) | 181 (65) | 170 (69) | 171 (67) | 204 (66) | 210 (67) |
| Education >= secondary school *^(f)^* | 258 (70) | 192 (69) | 169 (68) | 172 (68) | 208 (68) | 214 (68) |
| **Health indicators and medical factors** |  |  |  |  |  | |
| BMI (kg m-^2^) | 26.6 (4.1) | 27 (4.2) | 26.1 (3.8) | 26.6 (4.1) | 26.7 (4.2) | 26.7 (4.2) |
| ApoE-ε4 carrier *^(g)^* | 70 (19) | 51 (18) | 45 (18) | 47 (18) | 55 (18) | 56 (18) |
| Regular physical exercise *^(h)^* | 100 (33) | 71 (30) | 72 (35) | 77 (36) | 88 (34) | 89 (34) |
| Hypertension *^(i)^* | 284 (77) | 206 (74) | 187 (76) | 194 (76) | 235 (76) | 242 (77) |
| Diabetes *^(j)^* | 36 (10) | 25 (9) | 23 (9) | 23 (9) | 31 (10) | 32 (10) |
| Hypercholesterolemia *^(k)^* | 223 (60) | 163 (58) | 153 (62) | 154 (60) | 179 (58) | 185 (59) |
| Antecedents of CVD *^(l)^* | 112 (30) | 80 (29) | 70 (28) | 79 (31) | 97 (31) | 99 (31) |
| **Medication** | |  |  |  | | |
| Antihypertensive medication use *^(m)^* | 216 (58) | 152 (54) | 136 (55) | 145 (57) | 178 (58) | 182 (58) |
| Diabetic medication use *^(n)^* | 27 (7) | 21 (8) | 14 (6) | 17 (7) | 21 (7) | 21 (7) |
| Lipid lowering medication use *^(o)^* | 130 (35) | 98 (35) | 88 (36) | 94 (37) | 111 (36) | 112 (36) |
| Psychotropics and antidepressant medication use *^(p)^* | 109 (29) | 84 (30) | 72 (29) | 67 (26) | 84 (27) | 86 (27) |
| Vitamin D supplement use | 15 (4) | 13 (5) | 7 (3) | 9 (4) | 14 (5) | 14 (4) |
| Values represent mean (SD) or N (%) of non-missing values. All variables except age and BMI are categorical (yes/no) and represent ‘yes’ category.  **(a)** Neurogenesis-centred biological susceptibility was based on previous work [22,23] and operationalised as a dichotomous variable by median split. **(b)** Participants were classified as either cognitively stable or with accelerated cognitive decline based on their average performance in five neuropsychological tests (i.e., the Mini-Mental State Examination, the Benton Visual Retention Test, the Isaac’s Set Test, and the Trail-Making Test part A and part B across five follow-up visits across the 12-year study duration [5]. **(c)** Alzheimer’s Disease (AD) cases included all diagnoses of probable AD, possible AD, and mixed dementia and was established and validated by an independent committee of neurologists. **(d)** Vascular and other dementias (VoD) included all diagnoses of vascular dementia, Parkinson dementia, lewy body dementia and frontotemporal dementia and was established and validated by an independent committee of neurologists. **(e)** Assessed using the Center for Epidemiological Studies-Depression scale (CES-D) [17,19]. CES-D scores ≥ 17 for men and ≥ 23 for women were used as indicators of a high depressive symptomatology. **(f)** Education was based on the highest level of attainment and considered dichotomously: either as no or primary level education only or as secondary/high school level and above. **(g)** ApoE genotype was considered dichotomously: presence of at least one ε4 allele. **(h)** Practice and intensity of physical exercise was assessed using a physical activity questionnaire for the older adults [24]. Regular exercise was classified as doing sport regularly or having at least one hour of leisure or household activity per day; described in detail in [25]. **(i)** Blood pressure ≥ 140/90 mmHg or antihypertensive medication use. **(j)** Glucose ≥ 7.2 mmol/L or antidiabetic medication use. **(k)** Fasting plasma total cholesterol ≥ 6.2 mmol/L or lipid-lowering medication use. **(l)** History of cardiovascular or cerebrovascular disease. **(m)** Includes all antihypertensive drugs, calcium channel blockers, diuretics, beta-blockers, and drugs acting on the renin-angiotensin system. **(n)** Includes all antidiabetic drugs except insulin. **(o)** Includes all statins, fibrates, or bile acid sequestrants. **(p)** Includes all psycholeptics and psychoanaleptics – antidepressants, psychostimulants, and nootropics.  Abbreviations: ApoE-ε4, allele ε4 for the apolipoprotein E gene; BMI, body mass index; CVD, cardiovascular disease; SOX2, SRY (sex determining region Y)-box 2; CC3, cleaved caspase 3; MAP2, microtubule-associated protein 2; SD, standard deviation. | | | | | | |

| **Table A.2. Interaction analyses between dietary and nutritional factors and neurogenesis-centred biological susceptibility status on cognitive decline, dementia, and depressive symptomatology using multivariable-adjusted logistic regression models** | | | | |
| --- | --- | --- | --- | --- |
| **Interaction between hippocampal neurogenesis (HN) and dietary/nutritional factors on outcomes** | **Cognitive Decline *^(a)^*** | **Incident Dementia: Alzheimer’s Disease *^(b)^*** | **Incident Dementia:**  **Vascular and other dementias *^(c)^*** | **Incident Depressive symptomatology *^(d)^*** |
|  | *FDR-adjusted p value* ***^(e)^*** | *FDR-adjusted p value* ***^(f)^*** | *FDR-adjusted p value* ***^(g)^*** | *FDR-adjusted p value* ***^(h)^*** |
| **Fatty acid biomarkers (% of total fats in plasma; n=279):** | | | | |
| HN*Myristic Acid | 0.82 | 0.90 | 0.10 | 0.10 |
| HN*Stearic Acid | 0.83 | 0.14 | 0.96 | 0.33 |
| HN*Palmitic acid | 0.94 | 0.71 | 0.18 | 0.54 |
| HN*Palmitoleic acid | 0.13 | 0.95 | 0.14 | 0.82 |
| HN*Oleic Acid | 0.75 | 0.49 | 0.50 | 0.16 |
| HN*Linoleic acid | 0.14 | 0.81 | 0.56 | 0.20 |
| HN*Gamma-linoleic acid (GLA) | 0.73 | 0.58 | 0.56 | 0.63 |
| HN*Alpha-linoleic acid (ALA) | 0.14 | 0.79 | 0.20 | 0.12 |
| HN*Arachidonic acid (AA) | 0.11 | 0.50 | 0.62 | 1.0 |
| HN*Eicosapentaenoic acid (EPA) | 0.98 | 0.80 | 0.27 | 0.10 |
| HN*Docosapentaenoic acid (DPA) | 0.30 | 0.95 | 0.46 | 0.22 |
| HN*Docosahexaenoic acid (DHA) | 0.60 | 0.45 | 0.40 | 0.81 |
| **Vitamin D and overall nutritional status biomarkers (n=247):** | | | | |
| HN*Plasma prealbumin levels (g‎/l) | 0.41 | 0.76 | 0.09 | 0.27 |
| HN*Plasma 25(OH)-vitamin D (ng‎/ml) | 0.55 | 0.18 | 0.55 | 0.25 |
| **Carotenoid and Vitamin E biomarkers (n=255):** | | | | |
| HN*alpha-carotene (µg/L) | 0.56 | 0.39 | 0.86 | 0.14 |
| HN*beta-carotene (µg/L) | 0.88 | 0.69 | 0.37 | 0.25 |
| HN*alpha-tocopherol (mg/L) | 0.78 | 0.16 | 0.10 | 0.59 |
| **HN*gamma-tocopherol (mg/L)** | 0.90 | 0.24 | 0.19 | **0.04*** |
| HN*beta-cryptoxanthin (µg/L) | 0.22 | 0.91 | 0.41 | 0.51 |
| HN*zeaxanthin (µg/L) | 0.71 | 0.35 | 0.53 | 0.98 |
| HN*lutein (µg/L) | 0.35 | 0.28 | 0.92 | 0.45 |
| **HN*lycopene (µg/L)** | 0.75 | **0.01*** | 0.71 | 0.96 |
| HN*retinol (µg/L) | 0.18 | 0.26 | 0.89 | 0.36 |
| **Food consumption (serving per week, n=308) *^(i)^*:** | | | | |
| HN*Raw vegetables and salad | 0.42 | 0.55 | 0.34 | 0.20 |
| HN*Cooked vegetables (including soup) | 0.40 | 0.39 | 0.95 | 0.44 |
| HN*Pasta | 0.90 | 0.99 | 0.93 | 0.35 |
| HN*Rice | 0.64 | 1.0 | 0.50 | 0.81 |
| HN*Potatoes | 0.43 | 0.70 | 0.12 | 0.31 |
| HN*Legumes | 1.0 | 0.90 | 0.16 | 0.23 |
| HN*Fruits (including fresh, stewed, and juiced) | 0.91 | 0.52 | 0.52 | 0.38 |
| HN*Eggs | 0.13 | 0.75 | 0.90 | 0.84 |
| **HN*Poultry** | 0.09 | **0.02*** | 0.88 | 0.62 |
| **HN*Red meat** | 0.42 | **0.008**** | 0.79 | 0.90 |
| HN*Fish and seafood | 0.82 | 0.28 | 0.51 | 0.96 |
| HN*Sandwiches and pizzas | 0.12 | 0.78 | 0.38 | 0.46 |
| HN*Charcuterie | 0.56 | 0.27 | 0.46 | 0.70 |
| HN*Biscuits and cakes | 0.14 | 0.34 | 0.95 | 0.27 |
| HN*Sweets, sweetened beverages | 0.26 | 0.14 | 0.24 | 0.71 |
| HN*Dairy products | 0.42 | 0.11 | 0.69 | 0.25 |
| HN*Cereals (including bread) | 0.30 | 0.17 | 0.18 | 0.54 |
| HN*Tea | 0.64 | 0.64 | 0.92 | 0.35 |
| HN*Coffee | 0.37 | 0.14 | 0.10 | 0.62 |
| HN*Mediterranean diet score *^(j)^* | 0.66 | 0.86 | 0.67 | 0.14 |
| HN*Energy intake (calories per day) *excluding alcohol intake* | 0.23 | 0.38 | 0.13 | 0.16 |
| **Nutrient intakes (n=315) *^(k)^*:** | | | | |
| HN*Total carbohydrates (g/day) | 0.47 | 0.49 | 0.88 | 0.44 |
| HN*Total fats (g/day) | 0.49 | 0.84 | 0.34 | 0.65 |
| HN*Total proteins (g/day) | 0.59 | 0.88 | 0.93 | 0.95 |
| HN*Simple carbohydrates (g/day) | 0.24 | 0.10 | 0.74 | 0.55 |
| HN*Complex carbohydrates (g/day) | 0.32 | 0.59 | 0.45 | 0.77 |
| HN*Monounsaturated fats (g/day) | 0.26 | 0.33 | 0.83 | 0.58 |
| HN*Polyunsaturated fats (g/day) | 0.35 | 0.56 | 0.32 | 0.66 |
| HN*Saturated fats (g/day) | 0.18 | 0.29 | 0.97 | 0.73 |
| HN*Animal proteins (g/day) | 0.47 | 0.71 | 0.45 | 0.82 |
| HN*Vegetable proteins (g/day) | 0.68 | 0.72 | 0.85 | 0.11 |
| HN*Fibre (g/day) | 0.45 | 0.80 | 0.43 | 0.73 |
| HN*Cholesterol (mg/day) | 0.29 | 0.10 | 0.84 | 0.46 |
| HN*Myristic acid | 0.21 | 0.29 | 0.25 | 0.95 |
| HN*Palmitic acid | 0.38 | 0.38 | 0.25 | 0.40 |
| HN*Palmitoleic acid | 0.74 | 0.60 | 0.13 | 0.88 |
| HN*Stearic acid | 0.65 | 0.58 | 0.87 | 0.95 |
| HN*Oleic acid | 0.49 | 0.27 | 0.77 | 0.68 |
| HN*Linoleic acid | 0.45 | 0.86 | 0.19 | 0.51 |
| HN*Alpha linoleic acid (ALA) | 0.56 | 0.34 | 0.77 | 0.56 |
| HN*Arachidonic acid (AA) | 0.90 | 0.13 | 0.61 | 0.73 |
| HN*Eicosapentaenoic acid (EPA) | 0.29 | 0.28 | 0.14 | 0.61 |
| HN*Docosahexaenoic acid (DHA) | 0.54 | 0.73 | 0.62 | 0.66 |
| HN*Other saturated fats | 0.20 | 0.52 | 0.77 | 0.92 |
| HN*Other monounsaturated fats | 0.97 | 0.97 | 0.66 | 0.83 |
| HN*Equivalent beta-carotene (µg/day) | 0.91 | 0.11 | 0.29 | 0.26 |
| HN*Retinol (µg/day) | 0.70 | 0.38 | 0.75 | 0.41 |
| HN*Vitamin B1 (mg/day) | 0.84 | 0.73 | 0.86 | 0.61 |
| HN*Vitamin B2 (mg/day) | 0.43 | 0.96 | 0.37 | 0.16 |
| HN*Vitamin B3 (mg/day) | 0.42 | 0.48 | 0.29 | 0.13 |
| HN*Vitamin B5 (mg/day) | 0.10 | 0.86 | 0.42 | 0.75 |
| HN*Vitamin B6 (mg/day) | 0.26 | 0.96 | 0.82 | 0.13 |
| HN*Vitamin B9 (µg/day) | 0.13 | 0.14 | 0.10 | 0.22 |
| HN*Vitamin B12 (µg/day) | 0.79 | 0.68 | 0.84 | 0.40 |
| HN*Vitamin C (mg/day) | 0.54 | 0.63 | 0.50 | 0.76 |
| **HN*Vitamin D (µg/day)** | 1.0 | 0.31 | **0.04*** | 0.20 |
| HN*Vitamin E (mg/day) | 0.76 | 0.64 | 0.27 | 0.23 |
| HN*Sodium (mg/day) | 0.70 | 0.84 | 0.10 | 0.47 |
| HN*Potassium (mg/day) | 0.16 | 0.37 | 0.30 | 0.56 |
| HN*Magnesium (mg/day) | 0.13 | 0.42 | 0.88 | 0.92 |
| HN*Phosphorous (mg/day) | 0.89 | 0.93 | 0.12 | 0.92 |
| HN*Calcium (mg/day) | 0.89 | 0.76 | 0.30 | 0.98 |
| HN*Iron (mg/day) | 0.71 | 0.93 | 0.23 | 0.59 |
| HN*Zinc (mg/day) | 0.59 | 0.45 | 0.64 | 0.87 |
| **HN** represents neurogenesis-centred biological susceptibility status, which was based on previous work [22,23] and operationalised as a dichotomous variable by median split. For CD, HN represents above/below median levels of hippocampal cell death during differentiation (%CC3-d). For AD, HN represents above/below median levels of hippocampal cell death during proliferation (%CC3-p). For VoD, HN represents above/below median levels of hippocampal progenitor cell integrity (%SOX2). For depressive symptomatology, HN represents above/below median levels of hippocampal cell differentiation (%MAP2). **Dietary and nutritional variables**: Three aspects of diet/nutrition were used to inform present analyses: (1) Food consumption (in servings per week; n=308), (2) nutrient intakes (n=315) and (3) concentrations of 12 fatty acid biomarkers (n=279), transthyretin and vitamin D biomarkers (n=247), 6 carotenoid and 3 vitamin E biomarkers (n=255). Nutritional biomarker concentrations were determined in total plasma at baseline. **(a)** Participants were classified as either cognitively stable or with accelerated cognitive decline based on their average performance in five neuropsychological tests (i.e., the Mini-Mental State Examination, the Benton Visual Retention Test, the Isaac’s Set Test, and the Trail-Making Test part A and part B) across five follow-up visits across the 12-year study duration [5]. **(b)** Alzheimer’s Disease (AD) cases included all diagnoses of probable AD, possible AD, and mixed dementia and was established and validated by an independent committee of neurologists. **(c)** Vascular and other dementias (VoD) included all diagnoses of vascular dementia, Parkinson dementia, lewy body dementia and frontotemporal dementia and was established and validated by an independent committee of neurologists. **(d)** Assessed using the Center for Epidemiological Studies-Depression scale (CES-D) [17,19]. CES-D scores ≥ 17 for men and ≥ 23 for women were used as indicators of a high depressive symptomatology. **(e)** Estimated using logistic regression controlling for age, sex, education, physical activity, APOe4 carrier status, diabetes, and antihypertensive medication use. **(f)** Estimated using logistic regression controlling for age, sex, education, age of dementia onset, physical activity, APOe4 carrier status and psychotropic medication use. **(g)** Estimated using logistic regression controlling for age, sex, education, age of dementia onset, physical activity, plasma cholesterol concentrations, plasma glucose concentrations, diabetes, hypercholesterolemia, antihypertensive medication use, diabetic medication use and psychotropic medication use. **(h)** Estimated using logistic regression controlling for age, sex, education, baseline depressive symptomatology, physical activity, and plasma glucose concentrations. **(i)** Food consumption was determined by the Food Frequency Questionnaire (FFQ) at the 2-year follow-up visit. **(j)** Using data from the FFQ, a Mediterranean diet score was generated by adding the scores for each food group considered to be part of the Mediterranean diet as previously described [4]. **(k)** Nutrient intakes were determined by a 24h dietary recall at the 2-year follow-up visit. * *p* < .05; ** *p* < .01. FDR alpha threshold 0.05.  Abbreviations: FDR, false discovery rate correction; HN, hippocampal neurogenesis; CC3, cleaved caspase 3; SOX2, SRY (sex determining region Y)-box 2; MAP2, microtubule-associated protein 2. | | | | |

**Figure A.1. Schematic overview of key risk (+) and protective (-) dietary and nutritional factors for dementia and depressive symptomatology in individuals with a neurogenesis-centred biological susceptibility**

**A. Schematic representation of dietary and nutritional factors associated with dementia in individuals with a neurogenesis-centred biological susceptibility. (i)** Increased plasma lycopene concentrations and red meat consumption were both associated with an increased risk for future Alzheimer’s disease (AD) in individuals with a neurogenesis-centred biological susceptibility, i.e., lower levels of hippocampal cell death during proliferation, while increased poultry consumption may be protective against developing future AD in this susceptible subgroup. **(ii)** Increased vitamin D consumption was associated with an increased risk for future vascular and other dementias (VoD) in individuals with a neurogenesis-centred biological susceptibility, i.e., lower levels of hippocampal progenitor cell integrity. **B. Schematic representation of nutritional factors associated with depressive symptomatology in individuals with a neurogenesis-centred biological susceptibility.** Increased plasma γ-tocopherol concentrations were associated with increased depressive symptomatology in individuals with a neurogenesis-centred biological susceptibility, i.e., higher levels of hippocampal cell differentiation. Key: (+) represents a significant positive association; (-) represents a significant negative association. **Image created using BioRender. software.**

**REFERENCES**

**1**. Helmer C, Bricout H, Gin H, Barberger-Gateau P. Macronutrient intake and discrepancy with nutritional recommendations in a group of elderly diabetic subjects. Br J Nutr 2008; 99: 632–638.

**2**. Larrieu S, Letenneur L, Berr C *et al.* Sociodemographic differences in dietary habits in a population-based sample of elderly subjects: the 3C study. J Nutr Health Aging 2004; 8: 497–502.

**3**. Féart C, Jutand MA, Larrieu S *et al.* Energy, macronutrient and fatty acid intake of French elderly community dwellers and association with socio-demographic characteristics: data from the Bordeaux sample of the Three-City Study. Br J Nutr 2007; 98: 1046–1057.

**4**. Féart C, Samieri C, Rondeau V *et al.* Adherence to a Mediterranean Diet, Cognitive Decline, and Risk of Dementia. JAMA 2009; 302: 638–648.

**5**. Low DY, Lefèvre-Arbogast S, González-Domínguez R *et al.* Diet-Related Metabolites Associated with Cognitive Decline Revealed by Untargeted Metabolomics in a Prospective Cohort. Mol Nutr Food Res 2019; 63: 1–10.

**6**. Folstein MF, Folstein SE, McHugh PR. ‘Mini-mental state’. A practical method for grading the cognitive state of patients for the clinician. J Psychiatr Res 1975; 12: 189–198.

**7**. Goodglass H, Kaplan E. Assessment of Cognitive Deficit in the Brain-Injured Patient BT - Neuropsychology. In: Gazzaniga MS, editor. Boston, MA: Springer US, 1979: 3–22.

**8**. Isaacs B, Kennie AT. The Set test as an aid to the detection of dementia in old people. Br J Psychiatry 1973; 123: 467–470.

**9**. Reitan RM. Validity of the Trail Making Test as an Indicator of Organic Brain Damage. Percept Mot Skills 1958; 8: 271–276.

**10**. Bell CC. DSM-IV: Diagnostic and Statistical Manual of Mental Disorders. JAMA 1994; 272: 828–829.

**11**. Vascular factors and risk of dementia: design of the Three-City Study and baseline characteristics of the study population. Neuroepidemiology 2003; 22: 316–325.

**12**. KATZ S, FORD AB, MOSKOWITZ RW, JACKSON BA, JAFFE MW. STUDIES OF ILLNESS IN THE AGED. THE INDEX OF ADL: A STANDARDIZED MEASURE OF BIOLOGICAL AND PSYCHOSOCIAL FUNCTION. JAMA 1963; 185: 914–919.

**13**. Lawton MP. Scales to measure competence in everyday activities. Psychopharmacol Bull 1988; 24: 609–614.

**14**. Rosow I, Breslau N. A Guttman health scale for the aged. J Gerontol 1966; 21: 556–559.

**15**. McKhann G, Drachman D, Folstein M, Katzman R, Price D, Stadlan EM. Clinical diagnosis of Alzheimer’s disease: report of the NINCDS-ADRDA Work Group under the auspices of Department of Health and Human Services Task Force on Alzheimer’s Disease. Neurology 1984; 34: 939–944.

**16**. Román GC, Tatemichi TK, Erkinjuntti T *et al.* Vascular dementia: diagnostic criteria for research studies. Report of the NINDS-AIREN International Workshop. Neurology 43 1993 250–260.

**17**. Radloff LS. The CES-D Scale: A Self-Report Depression Scale for Research in the General Population. Appl Psychol Meas 1977; 1: 385–401.

**18**. BERKMAN LF, BERKMAN CS, KASL S *et al.* DEPRESSIVE SYMPTOMS IN RELATION TO PHYSICAL HEALTH AND FUNCTIONING IN THE ELDERLY. Am J Epidemiol 1986; 124: 372–388.

**19**. Lyness JM, Noel TK, Cox C, King DA, Conwell Y, Caine ED. Screening for Depression in Elderly Primary Care Patients: A Comparison of the Center for Epidemiologic Studies—Depression Scale and the Geriatric Depression Scale. Arch Intern Med 1997; 157: 449–454.

**20**. Radloff LS, Locke BZ, Homme D, Bjectifs O. Risques psychosociaux : Center for Epidemiologic Studies-. 2011; 475–480.

**21**. Cosco TD, Lachance CC, Blodgett JM *et al.* Latent structure of the Centre for Epidemiologic Studies Depression Scale (CES-D) in older adult populations: a systematic review. Aging Ment Health 2020; 24: 700–704.

**22**. Du Preez A, Lefèvre-Arbogast S, Houghton V *et al.* The serum metabolome mediates the concert of diet, exercise, and neurogenesis, determining the risk for cognitive decline and dementia. Alzheimer’s & Dementia 2021 1–22 https://doi.org/10.1002/alz.12428.

**23**. Du Preez A, Lefèvre-Arbogast S, González-Domínguez R *et al.* Impaired hippocampal neurogenesis in vitro is modulated by dietary-related endogenous factors and associated with depression in a longitudinal ageing cohort study. Mol Psychiatry 2022;

**24**. Voorrips LE, Ravelli AC, Dongelmans PC, Deurenberg P, Van Staveren WA. A physical activity questionnaire for the elderly. Med Sci Sports Exerc 1991; 23: 974–979.

**25**. Dupré C, Bongue B, Helmer C *et al.* Physical activity types and risk of dementia in community-dwelling older people: the Three-City cohort. BMC Geriatr 2020; 20: 132.
